# COVID-19: Lessons for Chile

**DOI:** 10.5334/ijic.5571

**Published:** 2020-07-30

**Authors:** Osvaldo Artaza

**Affiliations:** 1 Faculty of Social Sciences and Health of the University of the Americas, CL

**Keywords:** Public health policies, health governance, primary health care, COVID-19 response

Santiago de Chile July 15, 2020

Dear IJIC editors

Chile is living its hardest days, pushing its systems of care to its limits. In the face of the pandemic, Chile has not performed well: to date, Chile officially has about 350 thousand confirmed COVID-19 cases and more than 7,300 deaths in a population of 18 million inhabitants. The factors that may have influenced this are multiple: there are enormous social inequalities and large population groups living in overcrowded, precarious conditions, who must mobilize to provide for themselves; the social and cultural changes generated by the neoliberal development model have created high levels of individualism and little capacity for a reduced and subsidiary state to guarantee essential social rights; the loss of credibility and trust in authorities and institutions has been aggravated by the social and political revolt that began in October 2019 and that Chile has not yet resolved, generating the pandemic only a parenthesis, which will undoubtedly be broken again, in a deeper way, by the economic crisis that will follow the health crisis; the biomedical and hospital-centric paradigm prevailing in the elites, the fragmentation and segmentation of the Chilean health system and the scant importance given to primary health care strategy (PHC), may be some of the possible factors. Chile is a country with great inequalities and its fragmented and segmented health systems replicate the inequitable ways in which society is organized. The pandemic has exposed the deep weaknesses of our health systems.

The pandemic highlights key aspects that we must consider and that can explain our failures. Among these: the poor ability of the government to give governance to a fast and effective response; a tendency to prioritise economic matters over the health of the population; the insufficient institutional strength of public health and the insufficient capacity to act integrally in the territories, identifying and containing; the level of social inequalities, the weight of the social determinants of health and the basic social support that society offers to its most vulnerable groups, in order to be able to withstand prolonged and effective quarantines; the insufficient role given to the primary health care strategy; the poor quality of the communication delivered to the population, through confused spokespersons and questioned for their lack of transparency and veracity; the lack of trust that the population has in its authorities and institutions and the inadequate capacity of people to act in an organized and disciplined way, with solidarity and a sense of common good; and finally, the insufficient quality and lack of transparency of information available for decision making. All of these factors may explain the Chilean results.

Unfortunately, in Chile, care services are disintegrated, and primary health care strategy continues to be invisible, given the prevailing biomedical and hospital-centric paradigm existing in the ruling elites. This results in the loss of valuable expertise at primary health care level making it difficult to act in an integrated way with the community and in the territories.

Without a doubt, the pandemic has exposed our current weaknesses, and these weaknesses are translated into many deaths that could have been avoided. Along with many changes that our people demand, important changes in our health systems are urgent, as well as great transformations in the global health field.

